# A Distributed Multi-Hop Intra-Clustering Approach Based on Neighbors Two-Hop Connectivity for IoT Networks

**DOI:** 10.3390/s21030873

**Published:** 2021-01-28

**Authors:** Mohamed Sofiane Batta, Hakim Mabed, Zibouda Aliouat, Saad Harous

**Affiliations:** 1 LRSD Laboratory, Computer Science Department, Ferhat Abbas University-Setif 1, 19000 Setif, Algeria; mohamedsofiane.batta@univ-setif.dz (M.S.B.); zaliouat@univ-setif.dz (Z.A.); 2 FEMTO-ST Institute/DISC, University of Bourgogne Franche-Comte, 25200 Montbeliard, France; hmabed@femto-st.fr; 3 College of Information Technology, United Arab Emirates University, 15551 Al-Ain, United Arab Emirates

**Keywords:** IoT, WSN, multi-hop clustering, distributed clustering, dynamic intra-clustering

## Abstract

Under a dense and large IoT network, a star topology where each device is directly connected to the Internet gateway may cause serious waste of energy and congestion issues. Grouping network devices into clusters provides a suitable architecture to reduce the energy consumption and allows an effective management of communication channels. Although several clustering approaches were proposed in the literature, most of them use the single-hop intra-clustering model. In a large network, the number of clusters increases and the energy draining remains almost the same as in un-clustered architecture. To solve the problem, several approaches use the k-hop intra-clustering to generate a reduced number of large clusters. However, k-hop proposed schemes are, generally, centralized and only assume the node direct neighbors information which lack of robustness. In this regard, the present work proposes a distributed approach for the k-hop intra-clustering called Distributed Clustering based 2-Hop Connectivity (DC2HC). The algorithm uses the two-hop neighbors connectivity to elect the appropriate set of cluster heads and strengthen the clusters connectivity. The objective is to optimize the set of representative cluster heads to minimize the number of long range communication channels and expand the network lifetime. The paper provides the convergence proof of the proposed solution. Simulation results show that our proposed protocol outperforms similar approaches available in the literature by reducing the number of generated cluster heads and achieving longer network lifetime.

## 1. Introduction

IoT networks cover progressively more aspects of our daily life and represent a convergence field of multiple technologies. The basic idea is to allow the variety of devices, or things, present around us (smartphones, sensors, cameras, laptops, Radio-Frequency Identification tags (RFID), etc.) to interact and cooperate to achieve a common goal and make users lives easier [1]. With extensive attention, IoT has rapidly spread to various fields of interest, i.e., smart healthcare, smart city, smart home, intelligent transportation systems and many other applications [2]. Such systems could be seen as a large number of heterogeneous devices that need to access and be accessible from the Internet [3]. This paradigm enables new technologies [4,5] such as heterogeneous types of big data, Cloud and Fog Computing, Data Centers, etc. IoT devices perform more functionalities than mere sensing, leading to a fast depletion of the available energy where battery recharging is often costly or economically disadvantageous. Therefore, energy efficiency is a basic concept that should be incorporated into the overall networks infrastructure [6].

**Figure 1:**
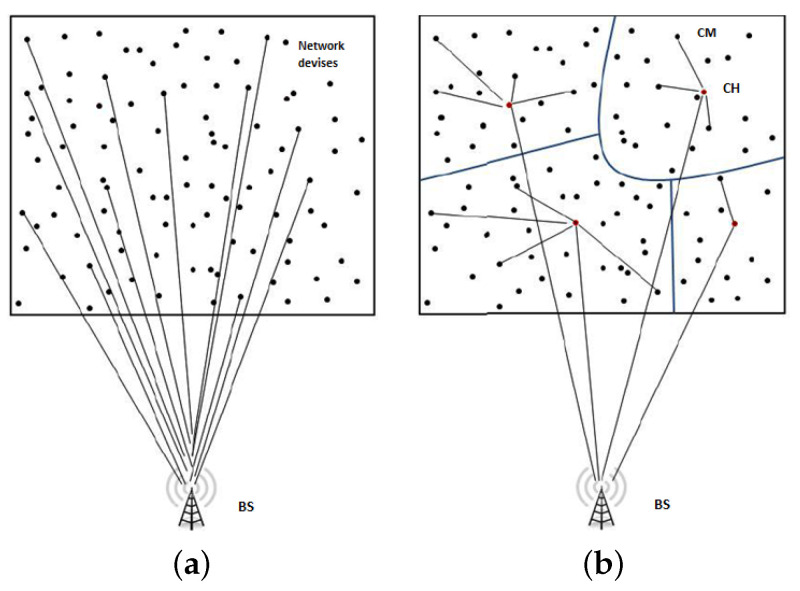
Connections toward the base station (**a**) using a flat architecture with individual connections, (**b**) using a structured and cluster-based architecture.

Network clustering is a technique that has been widely used in wireless networks. Indeed, grouping sensor nodes into clusters is an effective way to improve the network performance due to its ability to extend the networks lifetime [7,8] and increase the scalability of the network [9]. Figure 1 illustrates a simplified example of devices connecting with the Base Station (BS) using a flat architecture (Figure 1a) versus connecting through a cluster based scheme (Figure 1b). This figure shows how the number of direct communication channels with the BS can be considerably higher using a flat network. The clustering is used as a functional solution for managing the multi-channel communications [10]. Many channel management mechanisms were proposed to improve the radio capacity of the network [11,12]. However, in a flat and dense network, the base station may not satisfy the simultaneous connection resulting in a waste of wireless communication resources and energy.

Several previously proposed clustering protocols focus on the multi-hop inter-clustering [7,9,13] between the cluster heads (CH’s) and the base station to increase the network durability, however only few consider the intra-cluster communication (between devices and their CH). Usually, existing intra-clustering schemes [14,15,16] assume a direct connection between Cluster Members (CMs) and their CHs, therefore, a high number of clusters are formed. In a large scale network, the distance between nodes and their CHs may not be short enough for communication. Therefore, direct communication becomes obstructive and k-hop intra-clustering communication should be applied to ensure the network scalability. The k-hop intra-clustering model received a considerable interest from the research community [17,18,19] since it expresses (in a better way) the features of well-organized network. Indeed, k-hops intra-clustering model reduces the number of clusters which reduces the number of long-range communication channels and increases network durability [9]. The objective of k-hop clustering is to organize nodes into clusters where the path between cluster members and their corresponding cluster head is shorter than *k* hops of distance. It provides a robust topology in dynamic networks since cluster members may not be in direct contact with their CH, hence reconfiguration events (cluster head election and re-affiliations) are limited [20]. The maximum number of hops *k* inside each cluster could be easily determined according to the targeted application requirements and the deployment area’s nature. The proposed algorithm uses a weight based mechanism during the clustering process which consists of selecting the node with the maximum weight among its k-hop neighborhood as a CH. The weight of each node is based on three parameters, namely: the two-hop connectivity ratio, the remaining energy and the communication quality. A k-hop routing tree is elaborated within each cluster in a distributed fashion, where CMs use the Received Signal Strength Indicator (RSSI) to join the tree and select their parents by following the shortest routing path leading to the corresponding CH. Therefore, the k-hop clusters topology will further improve the energy efficiency and extend the network lifespan.

### 1.1. Network Architecture

**Figure 2:**
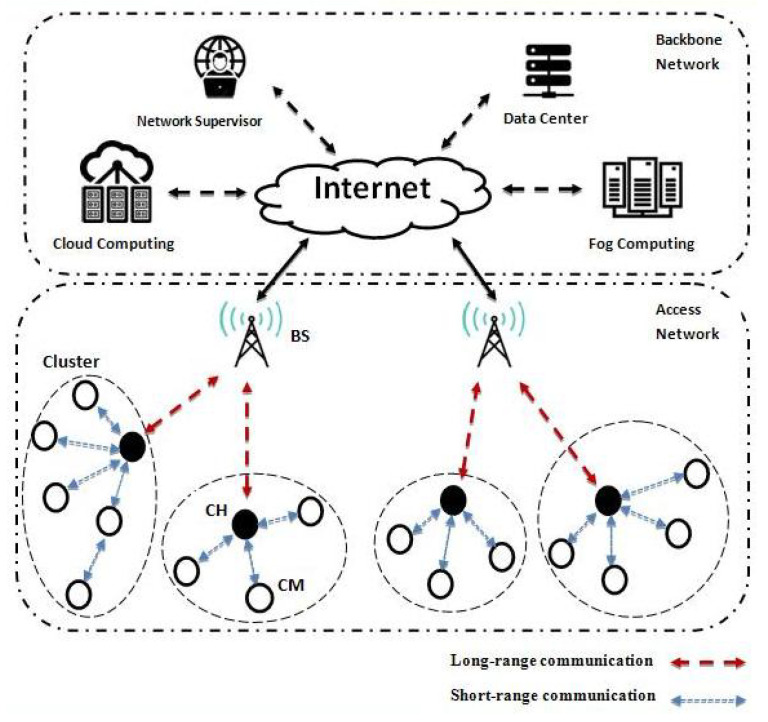
IoT network architecture.

The architecture of an IoT network is usually composed of two layers: the Access Network (AN) and the Backbone Network (BN). The AN contains two kinds of components (Figure 2): the Internet gateways (access points, base stations, NB, eNB, gNB [21], etc.) and a variety of deployed devices. The Base Stations (BS) acts as a gateway that allows network devices to connect with the Internet. On the other hand, the BN is mainly composed of the Internet that streams the collected data to a particular distant service (for network monitoring or saving and processing). In the k-hop intra-clustering, nodes of the same cluster exchange their information using a short-range communication. The IEEE 802.15.4 standard is commonly used for this purpose [22] and is adopted by many low-cost wireless interfaces such as Bluetooth [2], Zigbee [23], WirelessHART [24], 6LoWPAN [25], Z-Wave [26], etc. Where the energy consumption is reduced and devices can work for years without replacement. In contrast, to access the Internet getaway (typically the BS), network devices usually use long-range communication technologies [27], such as Wi-Fi (IEEE 802.11 [28]), LTE, LTE-M [29], etc. Long-range communication presents significantly high energy consumption and constraints devices lifetime to few days. The choice of the communication interface depends on the system application. Moreover, advanced communication technologies for Low Power Wide Area (LPWA) [30] were proposed in the literature to manage even wider communication areas, such as BLE (Bluetooth Low Energy) [31], WAVENIS [22], LoRaWAN (LoRa) [32] and Narrowband IoT (NB-IoT) [30]. Table 1 shows a brief comparison of some prevalent existing communication technologies with their associate application.

### 1.2. Motivation

As discussed in the introduction, when each network device is directly connected to the gateway, numerous energetic and radio channel access problems are faced, especially in dense and large networks [33]. Clustering the network by using direct connections among nodes and their CH engenders a high number of small clusters [34]. K-hop intra-clustering has demonstrated many advantages to avoid the congestion problems and prolong the network lifetime [5,35]. However, k-hop intra-clustering protocols are mostly centralized and only consider the node direct neighbors information, which lacks robustness. Indeed, with a centralized k-hop scheme, the BS is a central point of failure, i.e., a potential BS failure will obstruct the execution of the whole protocol. A loss of a critical node or a communication error could eventually cause a severe clustering failure because some data are usually of higher importance in a centralized approach. Moreover, when the network scales up, the BS may become a performance bottleneck. In this regard, this paper presents a new distributed k-hop intra-clustering protocol that takes into consideration the two-hop neighbors connectivity information. The main objective is to optimize the set of CHs in order to reduce the number of long-range communication channels to avoid congestion issues and extend the network lifetime.

The main contributions of this work are as follows:

- A new connectivity metric is introduced to elect the set of appropriate CHs. The novelty of this metric is taking into account the two-hop connectivity of the current node and its surrounding neighborhood (instead of using the traditional direct neighbors connectivity), in order to strengthen the clusters stability.
- The design of the algorithm is inspired from the distributed self-stabilizing systems. We prove that the algorithm converges within O(n+k) rounds, which represents the upper bound of the time complexity, *n* is the number of network nodes and *k* is the depth threshold of the clusters. This perspective allows network devices to efficiently tolerate potential failures that can occur locally in the dynamic topology.
- The proposed approach generates clusters with an energy efficient topology by reducing the distance between nodes and their respective CH. The adopted approach is peculiar in that it constructs the intra-cluster links in a distributed manner rather than using a centralized algorithm executed by the CH.

The remainder of this paper is structured as follows: Section 2 describes some related works. The network model and the algorithm objectives are presented in Section 3. Section 4 is dedicated to the presentation of our contribution. The energy model and transmission reliability are presented in Section 5. Section 6 is devoted to the convergence proof of the proposed scheme. The complexity of the algorithm is analyzed in Section 7. Section 8 describes the experimental settings and the simulation results. We conclude our work in Section 9.

## 2. Literature Review

Several researches focus on clustering techniques for wireless networks [8,20,22,36,37,38,39]. In this section, we present some of the prevalent one-hop and k-hop intra-clustering approaches designed for wireless networks (Table 2). Many researchers have addressed the one-hop intra-clustering [5,40,41,42] in order to design an energy efficient network. Low Energy Adaptive Clustering Hierarchy (LEACH) [43] is one of the first and most well-known energy efficiency clustering algorithms in this field. In LEACH, each sensor joins the closest one-hop away CH. The role of CH is rotated randomly and periodically over nodes to extend the network lifetime. This work has motivated many variations and improvements such as EP-LEACH [44], FL-LEACH [45] and MH-LEACH [46].

Xiao et al. [44] improved LEACH protocol by introducing the energy harvesting capability in the CHs election, which minimizes the energy consumption of devices. Authors in [45] proposed an amelioration of LEACH that shares the same heuristic and integrates a fuzzy inference system to determine the desired number of CHs in the network by only using the network density as an input. Chung-Wei [47] proposed a bio-inspired clustering algorithm. The idea is to use a multi-metric optimization algorithm to find a sub-optimal set of cluster heads and balance the number of CMs over clusters in order to reduce energy consumption.

The one-hop intra-clustering provides a convenient solution to structure the network. However, for large networks many clusters are formed. Therefore, isolated devices may appear and connectivity may be difficult to ensure. Multi-hop (or k-hop) intra-clustering alleviate the problem by producing a consistent structure in dynamic and large scale environments. MH-LEACH [46] or Multi-Hop LEACH is a novel extension of LEACH that supports the k-hop intra-clustering to increase energy efficiency. In this approach, cluster members send their data to the cluster heads through multi-hop communication and the CH aggregates data directly to the BS. The objective is to reduce the intra-cluster energy consumption by using multi-hop communications. However, in addition to the disadvantages of LEACH, this technique does not provide load balancing during the routing process.

E-PEGASIS [48] is a chain-based intra-clustering mechanism that aims to reduce the redundancy of data transmitted toward the BS. The algorithm finds a dominating set in the network and uses an ant colony optimization to elect the sub-optimal routing chain from the dominating nodes. This approach is an energy efficient solution. However, the time complexity required to organize the nodes into chain is costly. In [49], authors presented a k-hop Energy Constrained intra-clustering technique based on the Dominating Sets theory called K-ECDS. The proposed algorithm considers the energy limitation and models the problem of optimally choosing cluster heads based on the quality of communication channels and nodes cardinality. The clustering is distributed and aims to scale the network.

Authors in [14] proposed a new energy efficient hierarchical clustering using an intra-cluster communication scheme to improve the lifetime of the wireless networks. The proposed method uses an uneven clustering technique to alleviate the hot spot problem. The BS divides the network into three unequal clusters level, the CHs selection is based on the residual energy and the number of neighbours. The multi-hop intra-clustering is only applied in clusters located faraway from the BS. This protocol reduces the number of clusters and the messages overhead. However, the clustering is centralized, a potential BS failure will lead to the failure of the whole protocol. Authors in [19] presented a multi-hop intra-cluster clustering architecture. Initially, nodes enter a sleeping period and compute a wake up time slot according to their degree and the average distance to their neighbors. Nodes with a large set of neighbors and low average distance are awakened in an earlier execution time to be privileged for the CH election. Although the CHs selection provides more energy efficiency and balanced cluster formation, the clustering time is prolonged.

Mezghani [34] proposed a distributed multi-hop intra-clustering approach based on Khalimsky topology. The network is divided into k-hop large dynamic clusters. The CH election is distributed and weight based, the weight of each node is based on the residual energy, nodes degree and the neighbors communication probability. The node with the highest weight in its neighborhood is selected as a CH. The cluster topology is performed using the triangulation theory of Khalimsky to ensure optimal intra-clustering routing and reduce the energy consumption.

Traditional centralized intra-clustering algorithms often need knowledge of the entire network information. A loss of a critical node or a communication error could eventually cause a severe clustering failure because some data are usually of higher importance in a centralized approach. Therefore, our proposal is fully distributed allowing network nodes to act simultaneously and independently in order to make the network fault-tolerant.

## 3. Network Model and Algorithm Objective

In this study, the wireless network is assumed to be composed of a set of devices represented by a graph G=(V,E), where *V* is the set of network nodes and *E* is the set of edges that represents the communication and interfering links. $N_{k}\left(i\right)$ denotes the set of k-hop neighbors of the node i∈V, $N_{<k}\left(i\right)$ represents the set neighbors at distance <k-hop from *i*. N(i) denotes neighbors of node *i* at one hop. Deg(i)=|N(i)| is the degree of node *i*, i.e., number of nodes in the neighborhood of node *i*. The distance between the node *i* and the node *j* is Dist(i,j) (the number of hops in the shortest path between *i* and *j*). Nodes use multi-hop communication to relay their data toward their CH. We assume that all devices have the same transmission range (communication links are symmetric). Network devices use a single channel transceiver, i.e., they cannot receive from multiple senders or send and receive at the same time.

The objective of our proposed solution is to optimize the number of CHs and reduce the waste of energy caused by the long-range communication channels and decrease the risk of congestion. More precisely, we aim to find the smallest set *C* of selected cluster heads $CH_{n}\;(n\in \{1,2,\ldots ,|C|\})$ using a two-hop neighbors connectivity metric, while respecting a particular maximum hops constraint *k* between each node and its cluster head. Considering that $M_{n}$ is the set of nodes that belong to the cluster coordinated by the cluster head $CH_{n}$. The objective of the proposed mechanism is formulated as follows:

$$C=(Min\;|⋃CH_{n}\subseteq V\left|\right)\;\wedge \;\left(\;\forall i\in M_{n},\;\forall \;CH_{n}\in V\right):$$

$$Dist(i,CH_{n})\;is\;Minimized$$

where

$$M_{n}=\left\{\forall \;i\in V,\;\forall \;CH_{n}\in V\;|\;Dist\left(i,CH_{n}\right)\leq k\right\}$$

(1) $$\overset{\left|C\right|}{\underset{n=1}{⋃}}M_{n}=V\wedge \;\;\left(\forall \;n\neq m:M_{n}\cap M_{m}=\emptyset \right)$$

The communication over multi-hop short-range is usually more energy efficient than directly transmitting in a single-hop long-range communication [51]. Larger intra-cluster hops extends the coverage of the CHs, which reduces the number of elected CHs and the energy dissipation. However, in case of dense network, the interference rate will also increase, which expands the waste of energy due to collusion problems that may occur.

## 4. Proposed Approach

In this section, we present our distributed k-hop intra-clustering approach for wireless networks named Distributed Clustering based 2-Hop Connectivity (DC2HC). The clustering approach consists of grouping nodes with high connectivity into k-hop clusters. As cluster heads consume more energy compared with other nodes, the number of CHs should be reduced [49]. Therefore, DC2HC aims to produce an optimized number of clusters while maintaining the network coverage, reducing the number of isolated nodes and providing more extended network durability. The algorithm adopts a weight based mechanism during the cluster heads election process. This latter consists of selecting as a CH node with the maximum weight in its k-hop neighborhood. Multi-hop spanning trees [52] are formed inside each cluster where CMs use the Received Signal Strength Indicator (RSSI) to select their parents in the routing path leading to the corresponding CH. Therefore, clusters will be constructed with a topology that consumes less energy. Nodes weight combines three metrics:

1. **Two-hop connectivity ratio (TCR)**: this parameter represents the connectivity ratio of a node relative to its neighborhood. The TCR value of a given node *i* is calculated using Equation (2). Each node computes the average connectivity within its two hop neighborhood ($N_{\leq 2}\left(i\right)$) using $\Phi_{i}$, then compares the obtained value with the local degree to define the $TCR_{i}$. A negative $TCR_{i}$ value ($\left|N\left(i\right)\right|-\Phi_{i}\leq 0$) reflects the low connectivity proportion of the node *i* relatively to its surrounding environment. Higher $TCR_{i}$ value means that node *i* is surrounded by a large number of neighbors and these neighbors are well connected with many other nodes, thus *i* is a suitable CH candidate to maintain network connectivity. Therefore, it covers the largest number of nodes within the maximum hop constraint *k* and generates more fault tolerant and stable cluster topology. Indeed, in case of potential CH failure, the neighborhood of this node is well connected and the replacement of the current CH does not affect the cluster performance.
  $$\Phi_{i}=\frac{\left[\sum_{j=1}^{|N_{\leq 2}\left(i\right)|}\left|N\left(j\right)\right|\;/\;j\in N_{\leq 2}\left(i\right)\right]+\left|N\left(i\right)\right|}{|N_{\leq 2}\left(i\right)|+1}$$
  (2) $$TCR_{i}=\left|N\left(i\right)\right|-\Phi_{i}$$
2. **Residual energy ($E^{ratio}$)**: the remaining energy of network nodes is introduced in the CH election process. The ratio of remaining energy of a node *i* is computed as:
  (3) $$E_{i}^{ratio}=\frac{E_{i}^{residual}}{E_{i}^{init}}$$
  where $E_{i}^{init}$ is the initial energy of the current device and $E_{i}^{residual}$ is its remaining energy.
3. **Communication link quality (RSSI)**: DC2HC uses Radio Signal Strength Indicator (RSSI) as a metric to measure the quality of communications. The RSSI value (the received transmission power $P_{r}$) can be represented by the Log Distance Path Loss Model [53] as follows:
  (4) $$P_{r}\left(d\right)\left(\mathrm{dBm}\right)=P_{t}\left(\mathrm{dBm}\right)-10\times \alpha \;log\left(d\right)-X_{\sigma }$$
  $P_{t}$ represents the power of the transmitter’s radio signal in dBm. The distance *d* between the sender and the receiver is measured in meter. α is the path loss exponent that depends on the environmental conditions (α=2 in the free space propagation model). $X_{\sigma }$ is a Gaussian random variable used in case of shadowing effect. Otherwise, it equals zero.

Therefore, the weight $W_{i}$ of a node *i*, based on the previous parameters, is computed as follow:

(5) $$W_{i}=\alpha \times TCR+\beta \times E_{i}^{ratio}+\gamma \times RSSI$$

where α,β,γ represent the weighted coefficients of the corresponding metrics with the constraint α+β+γ=1. The contribution and importance of each parameter, relatively to the others, is indicated by its corresponding weighted coefficient. They are adjusted according to the system requirements and the network environment. A particular weight coefficient may be adjusted relatively to the others to obtain an optimal result for a particular network configuration. For example, in a low density environment, the residual energy should be favored. In case of a dense network, the connectivity should be favored. Whereas, in a noisy environment, the communication link quality needs to be considered. However, the proposed scheme is designed to work under a typical network with various configurations to cover different use-case scenarios. Therefore, in this experiment, all the weight coefficients are considered equal.

**Figure 3:**
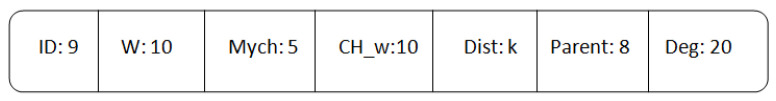
Example of Local State Values (LSV) structure.

In DC2HC, the clustering is completely distributed where each node has only a partial view of the network which consists of its two-hop neighbors knowledge. The algorithm is designed by a set of rules of the form [If condition then action], where the condition is a predicate defined over the node partial information. If the predicate is true, then the node is said to be enabled to make a move (execute an action). This algorithm structure is inspired from the self-stabilization algorithms that are considered as advantageous approaches for tolerating transient faults in a network [54], which is desirable in an environment with a dynamic topology. We assume that each node *i* has a unique identifier and maintains a set of variables that constitute the Local State Values of the node (LSV): node identifier ($ID_{i}$), its weight $W_{i}$, $Mych_{i}$ (the relative CH of the current node), $CH_{weight}$ (the weight of the relative CH), $Dist(i,Mych_{i})$ that indicates the distance to Mych (in term of hops), Deg(i), and its parent $P_{i}$ in the shortest data aggregation path. The structure of LSV is illustrated in Figure 3. Each node has a clustering record list CRL that contains a set of neighbors information required by the clustering process. Nodes store the received clustering information (LSV beacons) in this list.

Table 3 summarizes various notations used in the proposed approach. DC2HC is composed of three phases: the initialization phase, cluster heads election phase, and maintenance phase.

### 4.1. Initialization Phase

**Figure 4:**
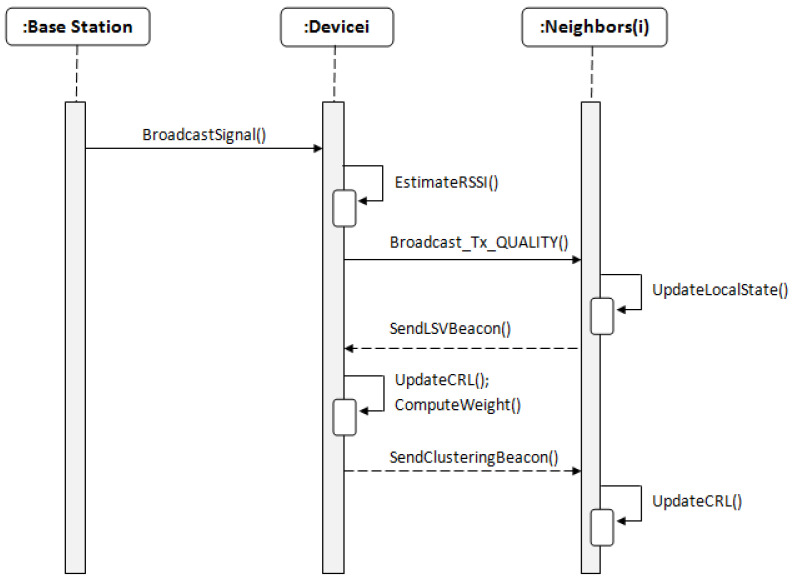
Sequence diagram of the initialization phase.

The BS broadcasts a periodic beacon signal at the initial stage. Based on the received signal, nodes can estimate the transmission quality to the BS (the RSSI value). After receiving this beacon, each node broadcasts a periodic Tx_QUALITY message in its transmitting range. After receiving a Tx_QUALITY message, a node updates its local state and replies by sending an LSV beacon. When the new node *i* receives LSV beacons from its neighborhood, it updates the $CRL_{i}$ list and computes its weight $W_{i}$ using Equation (5), then rebroadcasts a clustering message that contains the updated information. The LSV beacon is broadcasted every time that a local information changes, so that network nodes can maintain consistent and updated information about their local environment. The initialization process is described in Algorithm 1. Figure 4 summarizes the initialization phase.
**Algorithm 1** Initialization phase.Code for each node *i* **Variables**:$ID_{i}$: identifier$CRL_{i}=Null$$N_{i}=\emptyset$$Deg_{i}=0$$W_{i}=0$$RSSI_{i}=-100$ dBm **Upon** receiving BS signal:       Update RSSI value       Broadcast $Tx\_QUALITY_{i}$ message**Foreach** received LSV Beacon**do**:       $|N_{i}\left|=\right|N_{i}|+1$       Update $TCR_{i}$ value using Equation (2)       Deg(i)=|N(i)|       Update $CRL_{i}$ list**Endfor**    Update $W_{i}$ value using Equation (5)    Broadcast $LSV_{i}$ Beacon**Upon** receiving $Tx\_QUALITY_{j}$ message:       Send $LSV_{i}$ beacon to *j*

### 4.2. Cluster Head Election Phase

**Figure 5:**
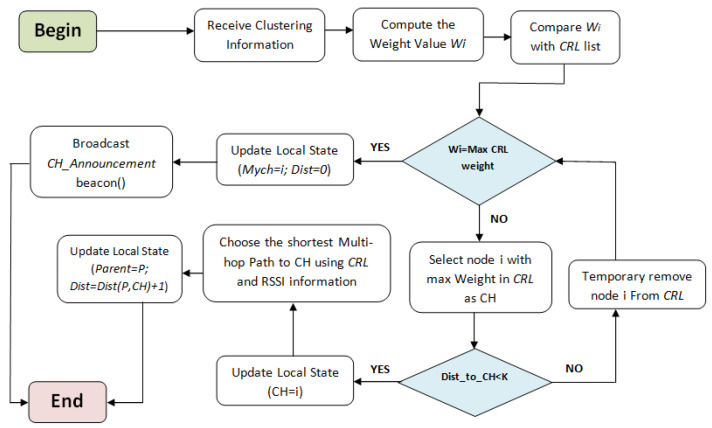
The cluster head election phase.

The pseudo-code of the clustering process is presented in Algorithm 2. We assume that no potential failure occurs during the execution of the CHs election phase. First, to elect a cluster head, a node *i* browses the $CRL_{i}$ list and compares its weight with the weight of its neighbors and with the weight of CHs that dominate its neighbors (rule R1). If $W_{i}$ is the greatest weight, *i* updates $Mych_{i}=ID_{i}$ (elect itself as a cluster head) and set $Dist(i,Mych_{i})=0$ (execute R2), then broadcasts a CH_Announcement beacon that contains the new CH information. Otherwise, *i* chooses the node with the highest weight value in $CRL_{i}$ list as its new CH. In the case where two or more nodes have the same highest weight, the node identifier is used as a tie-breaker, i.e., the device that has the highest ID is elected as CH. Rule R1 prevents the formation of poorly structured clusters, it allows network nodes to choose the most appropriate cluster head in their k-hop neighborhood. Otherwise, the node chooses the second weightiest node in $CRL_{i}$ and so on until finding the weightiest CH with less than *k* hops of distance. If no CH meets these requirements, then the current node elects itself as a new CH to avoid isolated node scenario. Node *i* updates $Mych_{i}$ with the identifier of the chosen node from (R1) and selects the path with the minimum number of hops toward the CH according to the information received from its one hop neighborhood (using rule (R3)). Consequently, nodes will be constrained to join the closest cluster that includes the weightiest CH, which prevents the formation of a height number of small clusters. The cluster heads election process is illustrated in Figure 5.
**Algorithm 2** Cluster Head election phase.Code for each node *i*  **Variables**:$ID_{i}$: identifierN(i): set of neighbors node of *i*$W_{i}$: weight of the node *i*Max_weight: temporary variable used to find the node with the highest weightOutput$CRL_{i}$: clustering record list of *i*$Mych_{i}$: the relative CH of *i*$Dist(i,Mych_{i})$: the minimal distance between *i* and its relative CH (in term of hop) **(R1): If**$Max\_weight\neq Max(\left\{CRL\left[ID_{j}\right]\left[W\right]\;|\;j\in \left(N\left(i\right)\vee Mych_{N(i)}\right)\wedge Dist\left(j,Mych_{j}\right)<k\right\}⋃\left\{W_{i}\right\})$**then**            $Max\_weight=Max(\left\{CRL\left[ID_{j}\right]\left[W\right]\;|\;j\in \left(N\left(i\right)\vee Mych_{N(i)}\right)\wedge Dist\left(j,Mych_{j}\right)<k\right\}⋃\left\{W_{i}\right\})$          $Mych_{i}=ID_{j}$         **Else**           **If** $i\neq j\wedge W_{i}=W_{j}$ **then**             **If** $ID_{i}\geq ID_{j}$                $Mych_{i}=ID_{i}$              **Else**                $Mych_{i}=ID_{j}$ **(R2): If**$\left(Mych_{i}=ID_{i}\right)\wedge CRL\left[Mych_{i}\right]\left[Dist_{i}\right]\neq 0$**then**            $Dist(i,Mych_{i})=0$          $CRL\left[Mych_{i}\right]\left[Dist_{i}\right]=0$          Broadcast CH_Announcement beacon **(R3): If**$\left(Mych_{i}\neq ID_{i}\right)\wedge \left(Dist\left(i,Mych_{i}\right)\neq Min\left(Dist\left(j,Mych_{j}\right)\;|\;j\in N\left(i\right)\wedge Mych_{i}=Mych_{j}\right)+1\right)$**then**            $Dist\left(i,Mych_{i}\right)=Min\left(Dist\left(j,Mych_{j}\right)\;|\;j\in N\left(i\right)\wedge Mych_{i}=Mych_{j}\right)+1$          $CRL\left[Mych_{i}\right]\left[Dist_{i}\right]=Dist\left(i,Mych_{i}\right)$

**Figure 6:**
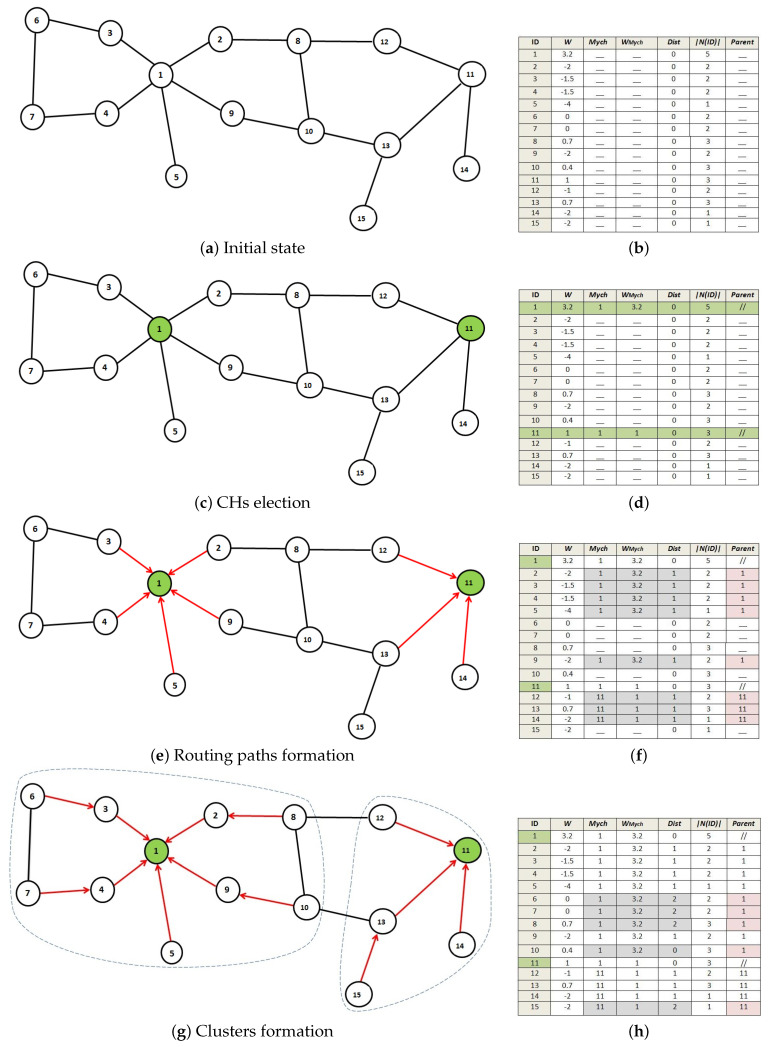
Execution scenario of Distributed Clustering based 2-Hop Connectivity (DC2HC) clustering process.

Figure 6 shows an execution scenario of the clustering process with k=2 in a small network (modeled by a graph). Green nodes represent the CHs and the white nodes represent the cluster members. Tables (b,d,f,h) in Figure 6 show the different nodes parameter. Figure 6a,g present, respectively, the initial network state and the final clustered network. Initially, network nodes have received the signal message from the BS and exchanged their clustering information. At each stage, the variables of each node used during the clustering are illustrated in the corresponding matrix. Notice in Figure 6a, the weight of nodes 1 and 11 are the highest among all the nodes in their CRL lists. During the following round (Figure 6c) these two nodes execute rule R1 to update their Mych value (became CHs) and rule R2 to update the distance $Dist(i,Mych_{i})=0$. Next, nodes 1 and 11 send an announcement beacon to their neighbors. When the neighbors of these nodes receive the CH_Announcement message, they update their CRL lists and join the CH with the highest weight using rule R1, then execute R3 to select the shortest path toward their CHs using neighbors information. At this stage, two clusters are formed in the network: C1{CH:1;CMs:2,3,4,9}, C2{CH:11;CMs:12,13,14}. Next (Figure 6e), as new members joined the cluster, the members send LSV message that contains the updated cluster information to their surrounding neighbors. Therefore, in the last state (Figure 6g) the same situation can be repeated for the rest of the nodes. The remaining unallocated nodes have received the clustering information and updated their CRL lists. These nodes join the cluster with the weightiest CH, then update their distance using R3 (Dist=2) to form the final two hops clusters.

### 4.3. Maintenance

In a dynamic wireless network, frequent topology changes can occur due to device mobility, battery draining, lack of coverage, etc. The clustered structure must be resilient to eventual node disconnections factor. Our approach adopts a continuous clustering maintenance, where devices periodically exchange control information. This mechanism is suitable for a dynamic scenario where disconnection events frequently occur [20]. The DC2HC re-clustering process can be locally driven and does not affect the entire network when disconnection events occur. Frequently updated control information enables a faster reaction to disconnection events. Thus, the clustered structure is more efficient and stable. The cluster maintenance phase is described as follows:

#### 4.3.1. Cluster Leaving

**Figure 7:**
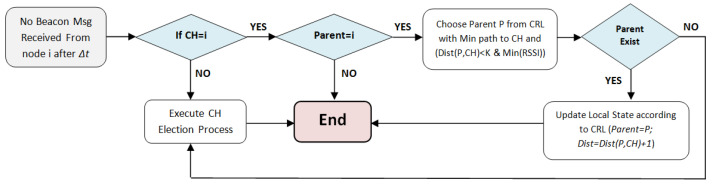
The cluster head leaving process.

Each node monitors its local environment through the periodically exchanged beacon messages to keep track of the neighborhood members. When a node leaves the cluster (following a disconnection factor), it stops transmitting; the surrounding neighbors detect this event and remove this node from their CRL lists. If the leaving node is a cluster head, rule (R1) will be activated and CMs re-execute this rule to elect a new CH and perform the necessary updates. Otherwise (the case where the leaving node is not a CH), rule (R3) will be activated and CMs having it as forwarder (to access the CH in multi-hop fashion) will choose another path to relay their information. The cluster leaving process is illustrated in Figure 7.

#### 4.3.2. Cluster Joining

When a node *i* decides to join a cluster, it executes the initialization phase and broadcasts a join request to the nearest neighbors. These neighbors reply by sending an $LSV_{i}$ beacon that contains the clustering information. The new node saves the received information in its $CLR_{i}$ list and computes its weight value $W_{i}$. If it has the largest weight among all the $CRL_{i}$ members, then it elects itself as the new CH and broadcasts an announcement message, so that the surrounding neighbors can perform the necessary changes (set the new node as their new CH). Otherwise, the new node joins the cluster of the weightiest CH in its k-hop neighborhood. Next, *i* selects the closest node to the CH (node with the lowest RSSI value) as its new parent to relay the collected data.

**Figure 8:**
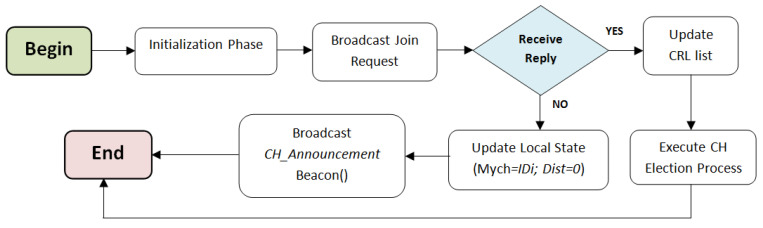
The cluster head joining process.

In the case where no reply packet is received, i.e., the new node is inside an isolated region, the new node *i* elects itself as a cluster head (to avoid isolated node scenario), updates its local state ($Mych_{i}=ID_{i},\;Dist\left(i,Mych_{i}\right)=0$) and broadcasts a CH_Announcement packet, since other nodes may have joined this region while *i* was executing the joining procedure. The cluster joining process is shown in Figure 8.

## 5. Energy Model and Transmission Reliability

**Figure 9:**
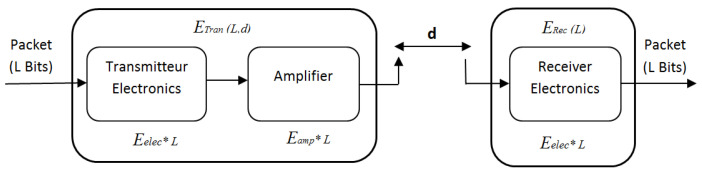
Radio energy consumption model.

Wireless network devices perform many functionalities (i.e., sensing, processing, transmitting and receiving information). Among all those functionalities, wireless communication is the one, which depletes most of the energy [36]. Therefore, in this study, we do not consider the energy dissipated in the data processing. The energy consumption model for the proposed DC2HC is adopted from the radio model used in [40], it assumes a simple communication model for the radio hardware battery consumption. In this model, the transmitter consumes an amount of power to run the radio electronics and the power amplifier. On the other side, the receiver consumes energy to detect and decode the radio signal as illustrated in Figure 9. Radio model parameters are illustrated in Table 4.

Equation (6) computes the energy required to transmit a data packet with *l* bits over a distance *d*, where $E_{elec}$ is the power dissipation to run the transmitter circuitry for transmission or reception. $\varepsilon_{amp}$ represents the power amplifier and depends on the distance to the receiver. $\varepsilon_{FS}$ is the power consumption of the free space propagation, $\varepsilon_{MFS}$ is the power consumption of multi-path propagation and $d_{0}$ is a threshold distance. When the distance between the transmitter and the receiver is lower than $d_{0}$, the free space model is used. Otherwise, the multi-path model is used. $d_{0}$ is calculated as:

$$d_{0}=\sqrt{\varepsilon_{FS}/\varepsilon_{MFS}}$$

(6) $$E_{Tran}\left(l,d\right)=E_{elec}\times l+\varepsilon_{amp}\times l$$

where:

$$\varepsilon_{amp}=\left\{\begin{array}{c} \varepsilon_{FS}\times d^{2}:d<d_{0}\\ \varepsilon_{MFS}\times d^{4}:d\geq d_{0} \end{array}\right.$$

Equation (7) represents the dissipated energy by the receiver in receiving ‘*l*’ bits of data.

(7) $$E_{Rec}=E_{elec}\times l$$

**Figure 10:**
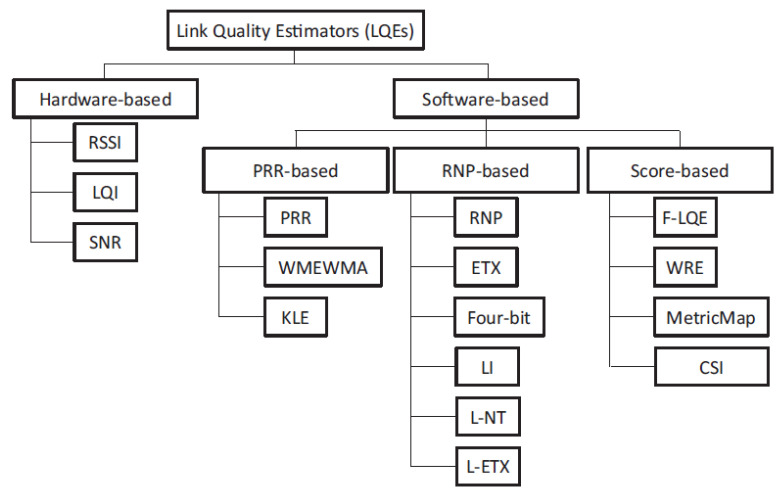
Available link quality estimators (LQE’s) [56].

Transmission reliability plays an essential criterion to improve the QoS of an application. The well-known available Link Quality Estimators (LQEs) are shown in Figure 10. Among all these estimators, Radio Signal Strength Indicator (RSSI), Link Quality Indicator (LQI) and Packets Received Rate (PRR) are the most common metrics used to estimate transmission quality [36]. RSSI measures the power signal of the received packets. LQI indicates the correctness of the received packets based on the first eight bits of that packet. LQI is an efficient estimator, but the RSSI perform good results with a small number of measurements and converge faster than LQI. PRR is considered as the best indicator for link quality [55]. However, it requires more time and energy to perform an accurate quality value. For these reasons, RSSI is chosen as the link quality estimator in this study. Lower RSSI value implies a weaker signal. It is measured in decibel, the closer this value is to zero the better the signal is. For example, −50 dBm is a good signal and −75 dBm is fairly reasonable.

## 6. Convergence

Since the network structure is dynamic, the clustering algorithms must have a quick convergence time (Convergence time: represents the duration from when a node starts the execution of the clustering algorithm until the cluster is finally constructed. At the convergence point, no more executions are performed by the node until the end of the clustering process). The convergence time of the algorithm is measured in terms of rounds. Each round is estimated by a number of movements. Therefore, in this section we will prove that our algorithm requires a finite number of movements in the clustering process, which implies its convergence. We assume that the network starts from an arbitrary configuration.

**Lemma** **1.** *Let i∈V be the node that has the highest weight value in G (i.e., $\forall j\in V-\left\{i\right\}:W_{i}>W_{j}\vee \left(\exists \;i\neq j:W_{i}=W_{j}\wedge ID_{i}>ID_{j}\right)$. i makes at most 2 movements (executes (R1) and (R3)).*

**Proof.**  We show that node *i* executes a finite number of movements. □

We assume that the value of $Mych_{i}$ is not updated. Since *i* is the weightiest node in *G*, it has the highest weight among all its neighbors ($\forall j\in N\left(i\right):W_{i}>W_{j}$). The rule R1 is enabled at node *i*, it will update the $Mych_{i}$ value (CH). Next, R2 is executed, *i* updates the distance $Dist(i,Mych_{i})=0$, afterward, no more rules are executed by node *i*, so it will not make another move. $Mych_{i}$ and $Dist(i,Mych_{i})$ will have the same values during the setup phase.

**Lemma** **2.** *Neighbors at distance <k from the node with the highest weight i∈G will execute a finite number of movements.*

**Proof.**  We show that the k-hop neighbors of *i* execute a finite number of movements (at most 2×k movements) before reaching the final state (Final state (or stable state): represents the state where the node has reached the convergence point. From this state, the node has a correct clustering variable (LSV) and all the algorithm rules are disabled for this node). □

After that, node *i* (the node with the highest weight) reaches a stable state (Lemma 1). Rule R1 and R3 will be enabled for its direct neighborhood j∈N(i), these neighbors will update their Mych value ($Mych=ID_{i}$), then execute R3 to update the distance Dist(i,j)+=1 (2 movements). After that, no more rule will be enabled in the neighborhood of *i* (N(i) has reached a final state).

This execution will be repeated by all nodes $\in N_{2}\left(i\right)$. Each node $j\in N_{2}\left(i\right)$ will execute at most 2 movements (R1 and R3) then finish their execution. Hence, the same scenario is repeated for the rest of nodes until it covers all the nodes in the sub-graph $G^{{}^{\prime }}=\left\{i⋃N_{\leq k}\left(i\right)\right\}$ that contains *i* and its k-hop neighborhood. Thus, the k-hop neighbors of the node with the highest weight reach the final state after at most 2×k movements and will not be able to execute any more movements.

**Lemma** **3.** *The stability of DC2HC is ensured.*

**Proof.**  Since node *i* with the highest weight in the network executes a finite number of movements (Lemma 1) and the k-hop neighbors of node *i* also execute a finite number of movements (Lemma 2). This implies that the sub-graph composed by the set of nodes $\left\{i\right\}⋃N_{k}\left(i\right)$ reaches a stable state after a finite number of movements. Let $G^{{}^{\prime \prime }}=\left\{G\;/\;\left(\left\{i\right\}⋃N_{k}\left(i\right)\right)\right\}$ be the graph obtained by removing the first stabilized sub-graph from *G*. The execution given above in *G* can be repeated in $G^{{}^{\prime \prime }}$, the second sub-graph that contains the node with the highest weight in $G^{{}^{\prime \prime }}$ stabilizes with the same reasoning, so all the nodes of the graph will follow this reasoning. This implies that the total number of moves executed by DC2HC is finite. □

## 7. Complexity

In the previous section, we demonstrated that the proposed approach terminates within a finite number of movements (2×(k+1)moves). However, the convergence complexity is not provided. In this section, the complexity of the proposed algorithm is examined in both theoretical and simulation-based analyses.

### 7.1. Theoretical Analysis

In the following, we assume the worst configuration from which our algorithm can start. Then we study the complexity of DC2HC.

**Figure 11:**
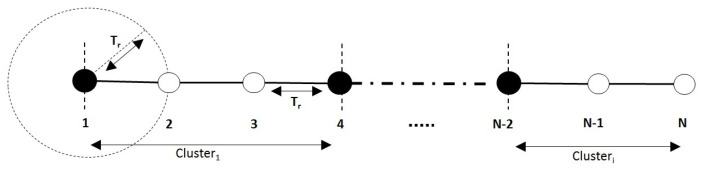
Worst-case scenario (k=2).

There is a known configuration from which DC2HC fails to provide good results (the worst-case scenario). This scenario is when nodes are related and organized in a straight line and their IDs are monotonically increasing or decreasing as illustrated in Figure 11. In this configuration (network with low density), the maximum degree of each node i∈G is: 0<Deg(i)≤2. In this case, network nodes will have the same weight and the election of cluster head depends on nodes IDs. Although, this configuration is unlikely to occur in a real-world network. It will allow the computation of the complexity in the worst case.

In the following, we prove that the time complexity of DC2HC is at most O(|V|+k) rounds, where |V| is the number of nodes on the network.

**Lemma** **4.** *Let i be the node with highest weight in G then:*

 *(a)* *After 2 rounds and in all following rounds, i is a cluster head ($Mych_{i}=ID_{i}$ and $Dist(i,Mych_{i})=0$).*
 *(b)* *After 2×k+2 rounds and in all following rounds, the neighbors of node i at distance ≤k form a cluster, where $\forall j\in N_{\leq k}\left(i\right):\left(Mych_{j}=i\right)\wedge \left(Dist\left(i,j\right)\leq k\right)$.*

**Proof.**  First, we demonstrate that (a) is true. In the first round, as *i* is the node with the highest weight in *G*, it has the high weight value among all its neighbors.

$$\forall j\in V-\left\{i\right\},\;z\in N\left(i\right):\left(W_{i}>W_{j}\right)\Rightarrow \left(W_{i}>W_{z}\right)$$

□

This node executes rule R1 to become a CH. In the second round, *i* executes rule R2 to update the distance value $Dist(i,Mych_{i})=0$. After that, no more rules are activated at node *i*, so it will not execute another move in the following rounds. Property (b) means that the sub-graph $G^{{}^{\prime }}=\left\{i⋃N_{\leq k}\left(i\right)\right\}$ that contains node *i* and its neighbors at distance ≤k reaches a stable state after at most 2+2×k rounds. In the 3rd round, when node *i* finishes its execution, rule R1 will be enabled for its neighborhood. These neighbors join the cluster of node *i*. Next, they execute R3 to update their distance $Dist(i,Mych_{i})=1$ (round 4). After round 4, no more rules can be activated in the neighborhood of *i*, so they will not execute other movements in the following rounds.

The argument given above is repeated for the neighbors at distance ≤k from *i* (i.e., neighbors of *i* will reach a stable state after 2×k rounds). Therefore, to form a cluster, two rounds are required to elect the CH and 2×k rounds to form the cluster. By induction, each node with the highest weight and its neighbors finish their execution after at most 2+2×k rounds.

**Lemma** **5.** *The maximum number of clusters that can be generated with DC2HC in the worst case configuration is 1+n/(k+1) where n=|V|.*

**Proof.**  DC2HC divides the graph *G* into several spanning trees where each tree has the node with the highest weight as its root (the cluster head) and its neighbors at a distance ≤k represent the rest of this tree (cluster). Therefore, the minimum number of nodes that can be in a cluster is k+1. In the worst-case scenario (the graph shown in Figure 11), a related graph can contain n/(k+1) clusters. As each cluster is represented by only one CH, the graph can contain at most n/(k+1) cluster heads. One is added to the previous threshold to cover the case where nmod(k+1)>0. Thus, we obtain $[\frac{n}{k+1}]+1$, which represents the maximum number of CHs formed by DC2HC in the worst-case configuration. □

**Lemma** **6.** *DC2HC algorithm converges after at most 2×(n+k+1) rounds.*

**Proof.**  According to Lemma 4, the subset that contains the node with the highest weight i∈G and its neighbors at a distance ≤k ($i⋃N_{\leq k}\left(i\right)$) stabilizes after at most 2×k+2 successive rounds. The same process is repeated in the graph $G^{{}^{\prime }}=\left\{G\;/\;i⋃N_{\leq k}\left(i\right)\right\}$. As *G* contains at most $[\frac{n}{k+1}]+1$ clusters (Lemma 5) and each cluster takes 2×k+2 rounds to reach a stable state. Thus, the proposed protocol requires at most ($\left[\frac{n}{k+1}\right]+1)\;\times \;\left(2\times k+2\right)\;=\;\left(\left[\frac{n}{k+1}\right]+1\right)\;\times \;2\left(k+1\right)=\;2\left(n+k+1\right)$ rounds to form all the clusters in the network. □

**Lemma** **7.** *DC2HC algorithm has a linear time complexity of O(n) rounds.*

**Proof.**  Based on Lemma 6, the upper bound of the time needed for the execution of DC2HC is 2×(n+k+1) rounds. Thus, it is obvious that the time complexity (convergence time) of the algorithm is O(|E|+k). As |E|≥k, the proposed algorithm converges after at most O(n) rounds. □

**Lemma** **8.** *DC2HC algorithm has a linear space complexity.*

**Proof.**  Each node has to maintain the 2-hop neighbors information in its CRL data structure. Therefore, the space complexity of each node will not increase as long as the local density remains constant. The space complexity of DH2HC is $O(d_{2})$, where $d_{2}$ represents the 2-hop local density. As $d_{2}\leq n$, the complexity of the proposed algorithm is linear, which is needed in a resource constrained environment. □

The number of nodes inside clusters depends upon the network density and the *k*-hop constraint. Indeed, in the case of complete graph, all nodes can be grouped within a singleton cluster. Hence, the maximum number of nodes in a cluster is n, where n is the number of nodes in the network. However, this situation may not often occur in real world networks. Therefore, by considering a related graph, the maximum number of clusters that can be generated by the proposal is n/(k+1) (Lemma 5), thus, the minimum limit of member inside each cluster is equal to $\frac{n}{n/(k+1)}=\frac{n(k+1)}{n}=k+1$.

### 7.2. Clustering Property

To prove that the proposed approach works properly. Clustering properties (safety and liveness) need to be satisfied.

#### 7.2.1. Safety Property

The safety property ensures that network nodes are grouped into clusters and each cluster has only one CH to avoid overlapping between clusters.

**Lemma** **9.** *The safety property is verified.*

**Proof.**  Each ordinary node elects the node with the highest weight among its multi-hop neighbors as its cluster head and the variable Mych holds only one value based on CRL list. Thus, a node can only belong to one cluster at a time and is covered by a unique CH. As a result, the safety property is satisfied. □

#### 7.2.2. Liveness Property

The liveness property ensures that the clustering progresses normally and reaches a final state after a finite time.

**Lemma** **10.** *The liveness property is satisfied.*

**Proof.**  According to Lemma 3 and Lemma 6, the proposed algorithm executes a finite number of movements and converges after at most n+k rounds. Hence, the liveness property is satisfied.

## 8. Simulation

### 8.1. Experimental Settings

The performances of the proposed approach are analyzed and compared using a simulator implemented in java using Java Universal Network/Graph (JUNG) [57] a Java based library that allows the modeling, analysis, and visualization of a wireless network as a graph. The network topology is composed of a variable number of nodes (δ∈[40,1300]) distributed across a square area of size $\Delta^{2}$= 1000 × 1000 m${}^{2}$. A random distribution is assumed to generate a random network topology, which is typically used in clustering approaches in the literature to approximate a real deployment scenario. Conventional sensor network usually uses wireless communication standard with low power consumption [58], such as IEEE 802.15.4 Zigbee (with a maximum transmitting distance of 100 m) or the 802.11n [28] (with a transmitting distance of 70 m). Therefore, based on the wireless communication standards used by regular wireless networks, in this experiment, we assume that network nodes have a transmission range of Tr=70 m. We use the classic Unit Disk Graph (UDG) connectivity model [59] where communication links are symmetric, i.e., a wireless communication link exists between two nodes *i* and *j* if they are within each other’s transmission range ($Dist\left(i,j\right)\leq Tr_{j}\wedge Dist\left(j,i\right)\leq Tr_{i}$). The energy model used is described in Section 5, node has 1 joule of initial energy and the size of a data packet is l=100 bytes. The k-hop clustering decreases the number of reconfiguration events that may occur inside the cluster [20]. Therefore, instead of using a periodic CH rotation for load balancing. In this study, the CH rotation occurs when the weight of the current CH is exceeded by one of its k-hop neighbors, which means that the current cluster has undergone a considerable topology changes. This mechanism avoids the quick alternation of CHs and further reduces the number of clustering messages generated. The experiment parameters are listed in Table 4. Increasing the number of communication hops inside each cluster generates long routing paths which may extensively increase the data transmission delay. Thereby, the performance of our clustering algorithm is analyzed by considering k∈{1,2,3}. The k-hop constraint ought to be specified depending on the application requirements to improve the network performance. The proposed scheme is experimented with varying maximum hop values and different nodes density to analyze the network performance under different scenarios.

In the following section, the performance of our approach DC2HC is compared with two ubiquitous and recent protocols that belong to the same family of multi-hop clustering. MH-LEACH [46] and Mezghani protocol [34] are both used for the k-hop intra-clustering and their primary objective is to reduce the waste of energy and the number of generated clusters. Owing to these characteristics, these protocols are selected for the performance comparison to our proposed protocol.

Five parameters are used for the performance analysis of the simulated protocols, namely, the cluster heads cardinality, the energy consumption, number of exchanged messages, the average network lifetime and the number of dead nodes. The same parameters are used to compare the three protocols. Each simulation result is the average of 10 measurements for each used metric with varying density and node distributions.

### 8.2. Experimental Results

#### 8.2.1. Cluster Head Cardinality

**Figure 12:**
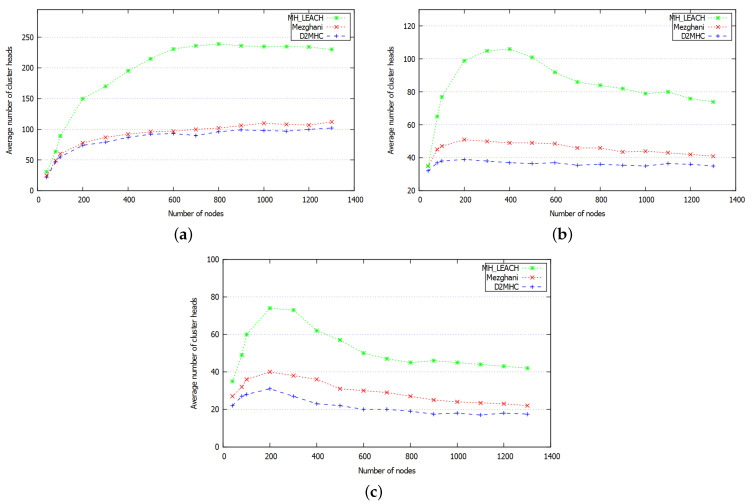
Average cluster head cardinality using the k-hop intra-clustering (**a**) k=1, (**b**) k=2, (**c**) k=3.

It represents the average number of generated clusters. This metric allows the evaluation of clustering efficiency. Low CHs cardinality reveals good characteristics of the clustering scheme as it represents the number of communication channels established with the BS. Moreover, reducing the CHs cardinality limits the usage of long-range communication channels which reduces the risk of congestion and improve the energy consumption. Figure 12 shows the average number of cluster heads generated by DC2HC, Mezghani [34] and MH-LEACH [46] protocols under different nodes density δ∈[40,1300]. We observe that MH-LEACH generates the highest cardinality. Indeed, MH-LEACH uses a probabilistic technique for load balancing the CH task among nodes. This technique does not consider the residual energy or the surrounding environment of nodes. When the density converges from 200 to 800 (δ∈[200,800]) with k∈{2,3}, the environment tends to be more connected which enlarges the set of neighbors within the transmitting range Tr of each node. Therefore, in Figure 12b,c, we observe that clusters cardinality of MH-LEACH start to decrease in the range between δ∈[400,800] because the coverage of CHs regroups more nodes. Mezghani protocol considers the residual energy of nodes which improves the cardinality when k=1 (improved by an average of 43.7% compared to MH-LEACH). However, the clustering process only considers the average one-hop degree of nodes, accordingly, when k∈{2,3}, the protocol slightly degrades in performance. The proposed scheme uses the k-hop clustering which minimizes the CHs cardinality and avoids having remote nodes within a forced singleton clusters. According to Figure 12, the proposed scheme shows better performance, it reduces the cardinality by an average of 18.3% and 62% when compared to Mezghani and MH-LEACH respectively. Table 5 shows the average gain of DC2HC compared to Mezghani and MH-LEACH in terms of CHs cardinality.

#### 8.2.2. Average Exchanged Messages and Consumed Energy

**Figure 13:**
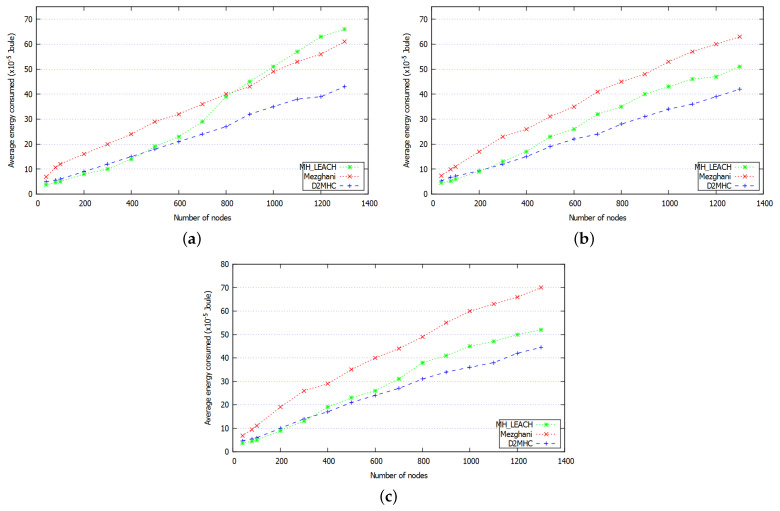
Average energy consumption of the three approaches with (**a**) k=1, (**b**) k=2, (**c**) k=3.

**Figure 14:**
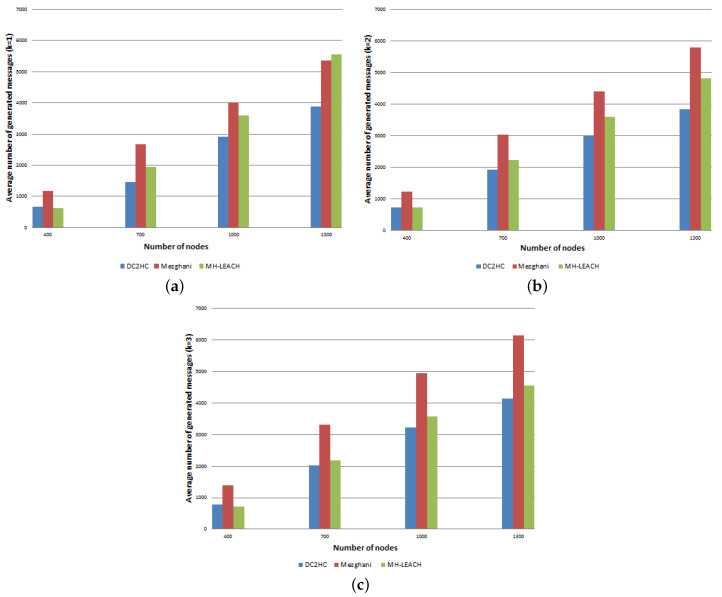
Average number of exchanged messages during the clustering process (**a**) k=1, (**b**) k=2, (**c**) k=3.

As wireless communication is the most expensive operation in wireless networks and depletes most of nodes energy, the number of exchanged messages and the consumed energy are strongly associated and have a significant impact on devices lifespan. Figure 13 and Figure 14, respectively, show the average energy consumed by network nodes and the average number of exchanged messages during the clustering process with according to nodes density increase and considering k∈{1,2,3}. The curves’ shape show that the energy consumed by network devices is proportional to the number of exchanged messages. In general, as the density increases the energy consumed and the number of messages, generated by the three approaches, increase as well. The multi-hop communications reduce the energy consumption by reducing the communication distance from CMs to their CH, especially when the size of the network scales. Figure 13 demonstrates that whatever the number of deployed nodes, DC2HC consumes less energy than the other protocols. This improvement can be attributed to the small number of elected CHs and reduced CH rotation that decreases the number of clustering messages exchanged. The number of exchanged messages is reduced by 36.5% and 6.9% compared to Mezghani protocol and MH-LEACH protocol respectively. The energy consumption is also reduced by an average of 21.7%, which is suitable for increasing the network lifetime. Table 6 summarizes the gains obtained by DC2HC when compared to Mezghani and MH-LEACH in terms of energy consumption.

#### 8.2.3. Average Network Lifetime

**Figure 15:**
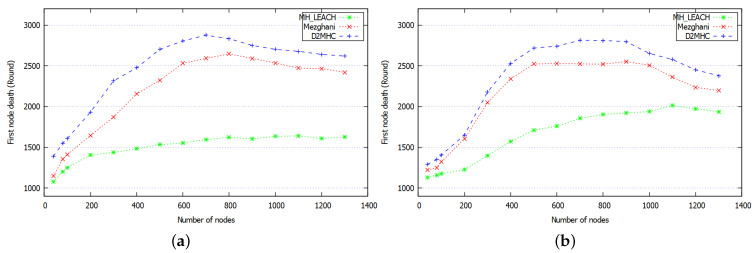
First node death according to different nodes density with (**a**) single-hop, (**b**) multi-hop.

Several works define the network lifetime as the period from network initialization to the round when the last node dies [9,46]. This parameter is important because it shows how long network nodes are able to run the protocol. Moreover, the early death of nodes can lead to a disconnection of some part of the network. Therefore, to evaluate the lifetime of the network, we considered the round at which the first node die (FND) and last node die (LND). Figure 15 shows the time at which the first node died according to different level of density. Typically, when the density increases, the connectivity among nodes increases, which reduces the CHs cardinality and balances the energy consumption among CMs. In MH-LEACH nodes die quickly because the protocol does not consider the residual energy of nodes. According to Figure 15, when the density is greater than 200 (δ≥200) the FND round of MH-LEACH tends to stabilize between 1400 and 1600 rounds. Mezghani protocol reveals better performance compared to MH-LEACH (improved by 45.2%) due to the reduced number of generated CHs (Figure 12) and to the usage of Khalimsky theory [34] to elaborate an energy efficient cluster topology. According to Figure 15a, DC2HC shows the highest durability, where the FND node reached 2876 round, whereas, in Mezghani and MH-LEACH it only reached 2591 and 1565 rounds, respectively. When increasing the maximum hop constraint (k>1), the performance of the three simulated protocols increase due to the usage of the multi-hop clustering, which reduces the energy consumption. The curve allure of DC2HC in Figure 15b shows an improvement in performance compared to the other approaches. Indeed, the proposed scheme generates a low cardinality of dense clusters based on the neighborhood TCR value. DC2HC uses the two hop neighbors information to favor nodes located in a well-connected region to be elected as a CH, it uses a multi-hop routing tree inside each cluster to improve the intra-cluster communications. Therefore, the connectivity among clusters is improved which reduces the waste of energy devoted to wireless transmission. Consequently, D2MHC achieves a gain factor of 13.3% and 58.5% compared to Mezghani and MH-LEACH respectively.

**Figure 16:**
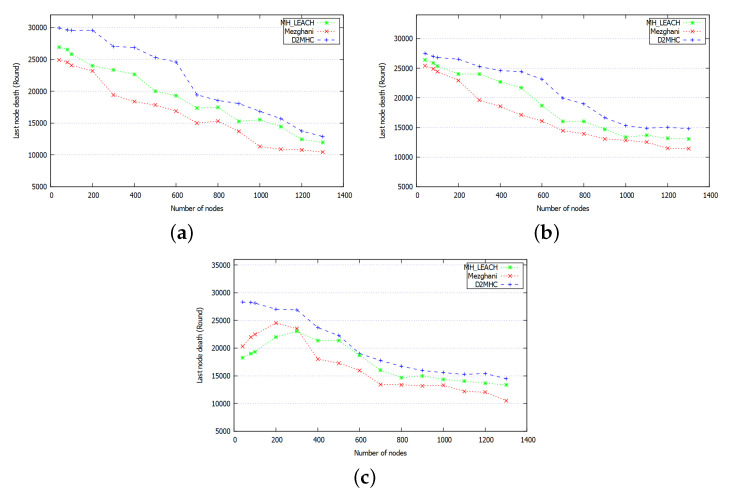
Last node death according to different nodes density with (**a**) k=1, (**b**) k=2, (**c**) k=3.

Figure 16 shows the round at which all network nodes died versus the network density (δ∈[40,1300]) considering k∈{1,2,3}. In MH-LEACH, the role of CHs is rotated randomly and periodically which reduces the number of setup messages, therefore, MH-LEACH behaves better than Mezghani protocol. However, using DC2HC allows a further extension of nodes lifetime since it minimizes the set of cluster heads and, thus, it reduces the waste of energy devoted to long range communications. Moreover, it limits the number of CH rotation which reduces the clustering messages and the overall energy consumption offering the network a longer lifetime. Our proposed protocol outperforms Mezghani and MH-LEACH protocols in terms of network lifetime by 26.3% and 13.3% respectively.

**Figure 17:**
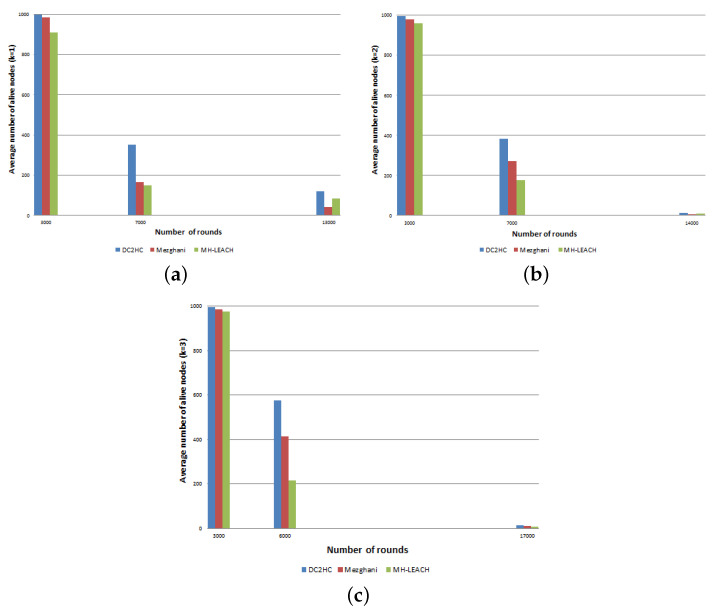
Average number of alive nodes over execution time with δ=1000 deployed nodes and (**a**) k=1, (**b**) k=2, (**c**) k=3.

Figure 17 depicts the total number of nodes that remain alive over execution time considering the number of deployed nodes δ=1000. Although the performances of the three protocols initially look similar, we observe that DC2HC has more alive nodes than the other algorithm with the growth of rounds. MH-LEACH selects the set of CHs based on random probabilities and does not consider nodes residual energy, which results in a faster death of network nodes. At the end of the clustering process only few nodes are remaining alive. Nevertheless, using our protocol allows network nodes to last for longer period. Whereas with the other protocols, nodes died faster. Table 7 summarizes the gains obtained by DC2HC when compared to Mezghani and MH-LEACH in terms of energy consumption.

## 9. Conclusions and Future Works

Energy consumption is a challenging design in the conception of wireless networks. Long distance communication channels used by cluster heads to reach the base station consume a significant amount of energy and accelerate the appearance of dead nodes and congestion problems. In this context, we present a new distributed approach for k-hop intra-clustering called Distributed Clustering based 2-Hop Connectivity (DC2HC) for large networks. The approach optimizes the set of representative cluster heads and extends the network lifetime. The cluster heads election is performed based on the two-hop neighbors connectivity of nodes to strength the cluster connectivity. It also considers the residual energy of nodes to balance the energy consumption among larger clusters. We proved that the proposed approach has a linear time complexity of O(|V|+k) rounds, where |V| is the number of nodes in the network. Various simulation experiments have been performed to evaluate the performance of the proposed scheme with different parameters. Simulation results show that the proposed algorithm provides better performance compared to similar algorithms. The cluster heads cardinality is reduced by 40.1%, the energy consumption is enhanced by 21.7% and the network lifetime is extended by 20.8%. As future work, we plan to study the effect of introducing the State of Health (SoH) of rechargeable batteries [60] during the clustering and integrating an optimized attack prevention schemes [61] to avoid the sudden death of network device and further increase the network durability.

## Figures and Tables

**Table 1:** Comparison of characteristics of different communicatioTn interfaces.

Protocol	Frequency Band	Data Rate	Range	Energy Consumption	Usage Area
Wi-Fi [28]	2.4/5.0 GHz	54 Mb/s	30 m	Medium	Home entertainment, Industrial
Bluetooth [2]	2.4 GHz	1 Mb/s	10 m	Low	Industrial, Traffic management
IEEE 802.15.4 [23]	868−915 MHz/2.4 GHz	250 kb/s	[10, 100] m	Low	Industrial, Traffic management, Smart home, vehicle monitoring
LTE [29]	2.6 GHz	10 Mb/s	≤15 km	High	Mobil telecommunications, Smart Cities
Wireless HART [24]	2.4−2.5 MHz	250 kb/s	[1, 100] m	Low	Healthcare, Industry automation
BLE [31]	2.4 MHz	1 Mb/s	200 m	Very Low	Healthcare, Home entertainment
Z-WAVE [26]	1 GHz	40 kb/s	30 m	Low	Home automation applications
WAVENIS [22]	865−916 GHz	100 kb/s	≤4 km	Very Low	Chemical and healthcare applications
LoRaWAN [32]	868−900 GHz	50 kb/s	≤15 km	Very Low	Smart City, industrial Monitoring, Agriculture
NB-IoT [30]	180 kHz	234.7 kb/s	≤35 km	Low	Industrial Monitoring, Smart City

**Table 2:** Clustering algorithms properties comparison.

Algorithm	Topology	Number of CH’s	Intra Clustering	Inter Clustering	Load Balancing	Energy Consideration	Benchmarks
LEACH [43]	Distributed	Undetermined	Single-hop	Single-hop	No	No	MTE
EP-LEACH [44]	Distributed	Undetermined	Single-hop	Single-hop	No	Yes (CH-election)	LEACH, TEEN
FL-LEACH [45]	Distributed	Determined	Single-hop	Single-hop	Yes	No	LEACH
Wu et al. [47]	Centralized	Undetermined	Single-hop	Single-hop	Yes	No	Not specified
MH-LEACH [46]	Distributed	Undetermined	Multi-hop	Single-hop	No	Yes (Multi-Hop transmission)	LEACH
E-PEGASIS [48]	Centralized	Determined	Multi-hop	Single-hop	No	No	PEGASIS, LBEERA
EE3C [50]	Centralized	Undetermined	Multi-hop	Multi-hop	Yes	No	Not specified
K-ECDS [49]	Distributed	Undetermined	Multi-hop	Multi-hop	No	No	ECDS, HEED
Singh et al. [14]	Centralized	Undetermined	Multi-hop	Multi-hop	Yes	Yes (CH-rotation)	EEUC, EUCA
Turgut [19]	Distributed	Undetermined	Multi-hop	Single-hop	Yes	No	Not specified
Mezghani [34]	Distributed	Undetermined	Multi-hop	Single-hop	Yes	Yes (intra-cluster routing)	MTE, HEED, APTEEN, EDC, THC, VCA

**Table 3:** Notation used in the proposed approach.

Symbol	Description
**$ID_{i}$**	Identity of device *i*
**$W_{i}$**	Weight of *i* (computed using Equation (5))
**$Mych_{i}$**	Relative Cluster Head of *i*
**$CH\_weight_{i}$**	Weight of $Mych_{i}$
**$Dist(i,Mych_{i})$**	Distance between *i* and $Mych_{i}$ (in term of hops)
**$P_{i}$**	Parent of *i* in the aggregation path toward the relative CH
**$Tr_{i}$**	Transmitting range of *i*
**Deg(i)**	Degree of *i* (Number of node in the neighborhood of *i*)
**$LSV_{i}$**	Local State Variable of *i*
**$CRL_{i}$**	Clustering Record List (Neighbors clustering information received)
**$TCR_{i}$**	Two-hop connectivity ration of *i*

**Table 4:** Simulation parameters used in the experiment setting.

Parameter	Value
Network size ($\Delta^{2}$)	1000 × 1000 m${}^{2}$
Node density	δ∈[40,1300]
Distribution of nodes	Random
connectivity model	Unit Disk Graph (UDG [59])
Transmitting range (Tr)	70 m
Maximum hop constraint k	{1,2,3}
α,β,γ	1/3, 1/3, 1/3
$E_{elec}$	50 nJ/bit
$\varepsilon_{FS}$	10 pJ/ bit/ M${}^{2}$
$\varepsilon_{Mfs}$	0.0013 pJ/ bit/ M${}^{4}$
Data packet size	100 bytes
Initial energy	1 Joule

**Table 5:** Synthesis of the average CH’s cardinality gains compared with Mezghani and MH-LEACH.

Clustering Algorithm	Intra-Cluster Topology		
	Single-hop	Two-hop	Three-hop
Mezghani	6.9%	21.1%	27%
MH-LEACH	55.3%	64.2%	67.3%

**Table 6:** Synthesis of energy consumption gains compared with Mezghani and MH-LEACH.

Clustering Algorithm	Intra-Cluster Topology		
	Single-hop	Two-hop	Three-hop
Mezghani	28.7%	37%	37.1%
MH-LEACH	10.6%	13.8%	3.1%

**Table 7:** Network lifetime gains compared with Mezghani and MH-LEACH.

Clustering Algorithm	Intra-Cluster Topology			
	**FND**		**LND**	
	Single-hop	Multi-hop	Single-hop	Multi-hop
Mezghani	30.5%	24.2%	16.4%	10.2%
MH-LEACH	14.4%	14.7%	75.1%	41.8%

## Data Availability

Not applicable.
